# Is speech function lateralised in the basal ganglia? Evidence from de novo Parkinson’s disease

**DOI:** 10.1136/jnnp-2024-334297

**Published:** 2024-09-17

**Authors:** Jan Rusz, Petr Dusek, Tereza Tykalova, Michal Novotny, Vojtech Illner, Michal Simek, Tomas Kouba, Petr Kryze, David Zogala, Evzen Ruzicka, Mário Sousa, Adriana Jorge, Tobias Nef, Paul Krack

**Affiliations:** 1 Department of Circuit Theory, Faculty of Electrical Engineering, Czech Technical University in Prague, Prague, Czech Republic; 2 Department of Neurology and Centre of Clinical Neuroscience, First Faculty of Medicine, Charles University and General University Hospital, Prague, Czech Republic; 3 Institute of Nuclear Medicine, First Faculty of Medicine, Charles University and General University Hospital, Prague, Czech Republic; 4 Movement Disorders Center, Department of Neurology, University Hospital of Bern, Bern, Switzerland; 5 ARTORG Center for Biomedical Engineering Research, University of Bern, Bern, Switzerland

**Keywords:** SPEECH, PARKINSON'S DISEASE, MOTOR CONTROL, MOVEMENT DISORDERS

## Abstract

**Background:**

Research on the possible influence of lateralised basal ganglia dysfunction on speech in Parkinson’s disease is scarce. This study aimed to compare speech in de-novo, drug-naive patients with Parkinson’s disease (PD) with asymmetric nigral dopaminergic dysfunction, predominantly in either the right or left hemisphere.

**Methods:**

Acoustic analyses of reading passages were performed. Asymmetry of nigral dysfunction was defined using dopamine transporter-single-photon emission CT (DAT-SPECT).

**Results:**

From a total of 135 de novo patients with PD assessed, 47 patients had a lower right and 36 lower left DAT availability in putamen based on DAT-SPECT. Patients with PD with lower left DAT availability had higher dysarthria severity via composite dysarthria index compared with patients with lower right DAT availability (p=0.01).

**Conclusion:**

Our data support the crucial role of DAT availability in the left putamen in speech. This finding might provide important clues for managing speech following deep brain stimulation.

## Introduction

 Asymmetry of motor symptoms associated with progressive asymmetric degeneration of nigral dopaminergic neurons represents a hallmark of Parkinson’s disease (PD).^1^ Although the precise mechanism behind this lateralisation of symptom onset is not fully understood, the side on which motor symptoms first appear may influence overall motor dysfunction. The distinctive motor changes in speech, characterised as hypokinetic dysarthria, are recognised as an early and common sign of the disease.^2^

However, there is a limited understanding regarding the potential impact of asymmetrical basal ganglia dysfunction on speech performance, despite ample evidence indicating that speech production in healthy individuals relies on the integrity and interaction of lateralised cortico-subcortical circuitries.^3^ The preliminary findings stem from small patient cohorts undergoing unilateral subthalamic deep brain stimulation.^4 5^ Specifically, stimulating the left subthalamic nucleus in patients with PD was associated with decreased articulatory accuracy and syllable rate, while stimulation of the right subthalamic nucleus had no impact on speech.^4 5^ In addition, a recent randomised trial showed that left subthalamic overstimulation might contribute to axial deterioration, including speech impairment, in patients with PD.^6^

Therefore, to disclose the suspected lateralised basal ganglia dysfunction on sensorimotor aspects of speech production, we aimed to objectively compare speech in de novo, drug-naive patients with PD with lower degrees of right and left dopamine transporter (DAT) availability in putamen defined using dopamine transporter single-photon emission CT (DAT-SPECT).

## Methods

### Study design

Between 2015 and 2024, we conducted prospective recruitment of de novo, drug-naive patients following the Movement Disorder Society (MDS) clinical diagnostic criteria for PD. The recruitment was part of a longitudinal project, ‘Biomarkers in PD (BIO-PD)’; the detailed protocol of this project has been described previously.^7^ Additionally, we recruited a group of sex-matched and age-matched healthy controls. Our exclusion criteria included a history of antiparkinsonian medication therapy, a history of communication disorders unrelated to parkinsonism (ie, problems in speech comprehension or expression) or other neurological disorders potentially affecting speech and a non-native Czech language speaker.

### Clinical evaluation

The evaluation of each subject included a structured clinical interview, history of drug and substance intake and current drug usage, clinical testing of motor symptoms using the MDS-Unified Parkinson’s Disease Rating Scale (MDS-UPDRS) part III, cognitive testing with the Montreal Cognitive Assessment (MoCA), autonomic testing with the Scales for Outcomes in Parkinson’s Disease-Autonomic Dysfunction (SCOPA-AUT) and handedness using the revised form of the Edinburgh handedness inventory (EHI). Perceptual speech severity was estimated using the speech item score from the MDS-UPDRS part III (ie, item 3.1). Symptom duration was estimated based on the self-reported first occurrence of PD motor symptoms.

### Asymmetry calculation via dopaminergic transporter imaging

In patients with PD, DAT-SPECT was performed using the [123I]−2-b-carbomethoxy-3b-(4-iodophenyl)-N-(3-fluoropropyl) nortropane (DaTscan, GE Healthcare) tracer following the procedure guidelines of the European Association of Nuclear Medicine and employing the common acquisition and reconstruction parameters described in detail previously.^8^ Semiquantitative analysis of images was performed using the DaTQUANT V2 software. The specific to non-displaceable binding ratios (SBRs) were determined in the bilateral putamen and caudate using the formula (nucleus uptake−background uptake)/background uptake with bilateral occipital lobes serving as the background reference region.

Similarly, as in previous studies (eg Lövdal *et al*^9^), we calculated the asymmetry index (AI) for both putamen and caudate according to the relative difference in SBR: AI = (SBR_right_–SBR_left_)/(SBR_right_+SBR_left_). To define patients with PD with the lower right (PD-Right) and lower left (PD-Left) DAT availability, we applied a cut-off value AI_putamen_=0.08 (ie, at least 8% difference between the right and left sides), corresponding to the upper quartile of the AI_putamen_ in DAT-SPECT of healthy controls with a similar average age of approximately 60 years from the Parkinson’s Progression Markers Initiative (PPMI) database.^10^

### Speech assessment

Speech recordings were performed in a quiet room with a low ambient noise level using a head-mounted condenser microphone (Beyerdynamic Opus 55, Heilbronn, Germany) placed approximately 5 cm from the subject’s mouth. Speech signals were sampled at 48 kHz with 16-bit resolution. All participants were instructed to read twice a short paragraph of standardised text composed of 80 words.

We performed an automated acoustic assessment of *imprecise consonants* via the duration of unvoiced stops (DUS), defined as the median duration of stop consonants. *Monopitch* was assessed via fundamental frequency variability (F0SD), defined as the SD of the fundamental frequency contour converted to a semitone scale. Finally, *prolonged pauses* were estimated via duration of pause intervals (DPI), defined as the median length of pause intervals.^2 11^ The values of variables were averaged across two repetitions of the reading passage to provide greater speech assessment stability. These variables were selected based on a recent multilanguage trial involving a large patient cohort of early-stage patients with PD that revealed imprecise consonants, monopitch and prolonged pauses as the most prominent speech patterns.^2^

Since the one-sample Kolmogorov-Smirnov test did not indicate non-normally distributed acoustic variables, these were first converted to the z-score using the mean and SD of the control group. As the main outcome, *dysarthria severity* via composite dysarthria index (CDI) was then generated by averaging these three z-scored variables. All analyses were performed in MATLAB (MathWorks, Natick, Massachusetts, USA).

### Statistical analysis

Speech differences between PD groups were calculated using an analysis of covariance. Associations between speech and asymmetry indexes were calculated by Pearson’s partial correlation. These analyses were adjusted by age, sex and MDS-UPDRS III. An independent sample t-test was used to compare the control and PD groups. A p value <0.05 was considered a threshold for statistically significant differences.

## Results

### Patients

A total of 135 de novo patients with PD and 70 healthy controls were assessed (table 1). According to the AI_putamen_ in DAT-SPECT, 83 patients (61%) had an asymmetric onset. From these, the right onset was detected in 47 patients (57%) and the left onset in 36 patients (43%), with only 8 patients with PD (10%) showing clinical symptoms on one side of the body (ie, lateralised MDS-UPDRS III on one side was equal to 0). According to the EHI, 81 patients with PD (98%) were right-handed; only one patient with left and one with right asymmetric onset were left-handed. No significant clinical differences, including sex proportion, age, symptom duration, MDS-UPDRS III, MDS-UPDRS III speech item, MoCA, SCOPA-AUT and EHI, were detected between PD-Right and PD-Left groups. Also, no significant difference between controls and any of the PD groups was seen for sex proportion, age, MoCA and EHI.

**Table 1:** Clinical characteristics= of participants

	PD	PD-Left	PD-Right	Controls	P value
	(n=135)	(n=36)	(n=47)	(n=70)	PD-Left versus PD-Right
Male	n=85	n=24	n=31	n=45	0.95#
	(63%)	(67%)	(66%)	(64%)	
Age (years)	60.5/12.2	57.6/13.2	59.3/11.6	60.9/11.4	0.53*
	(33–83)	(34–81)	(36–83)	(33–84)	
Motor symptom duration	2.0/1.9	2.1/1.5	1.8/1.9	–	0.40
(years)	(0.3–11.3)	(0.5–5.7)	(0.3–11.3)		
MDS-UPDRS III	29.7/11.8	29.4/9.8	27.0/10.1	4.2/3.7	0.27
	(6–70)	(6–49)	(10–53)	(0–17)	
MDS-UPDRS III speech item	0.65/0.49	0.67/0.48	0.60/0.50	0.09/0.28	0.52
	(0–2)	(0–1)	(0–1)	(0–1)	
MoCA	25.3/2.8	25.5/2.7	25.5/3.0	26.3/2.4	0.95*
	(17–30)	(17–30)	(18–30)	(19–30)	
SCOPA-AUT	9.3/5.8	8.6/6.2	8.9/5.4	6.2/4.5	0.83
	(0–34)	(0–34)	(0–21)	(0–24)	
EHI	82.0/35.7	88.4/26.8	80.1/31.6	86.3/30.8	0.21*
	(−100 to 100)	(−19 to 100)	(−75 to 100)	(−50 to 100)	
Putamen binding ratio left	1.50/0.41	1.34/0.32	1.75/0.42	–	<0.001
	(0.73–3.18)	(0.73–1.94)	(1.10–3.18)		
Putamen binding ratio right	1.44/0.37	1.73/0.39	1.28/0.30	–	<0.001
	(0.75–2.40)	(0.86–2.35)	(0.75–2.16)		
Asymmetric index putamen	0.03/0.12	−0.13/0.04	0.15/0.05	–	<0.001
	(−0.25 to 0.33)	(−0.25–0.08))	(0.08–0.33)		
Caudate binding ratio left	2.31/0.60	2.14/0.52	2.64/0.61	–	<0.001
	(1.01–4.00)	(1.01–3.06)	(1.35–3.73)		
Caudate binding ratio right	2.29/0.56	2.56/0.56	2.16/0.52	–	<0.001
	(0.93–3.86)	(1.05–3.55)	(0.93–3.17)		
Asymmetric index caudate	0/0.1	−0.09/0.05	0.11/0.06	–	<0.001
	(−0.24 to 0.27)	(−0.24 to 0)	(−0.02 to 0.27)		

Data are mean/SD (range) including p values between PD groups analysed using t-test.

* No significant difference between controls and PD-Left and PD-Right groups.

EHI, Edinburgh handedness inventory; MDS-UPDRS, Movement Disorder Society Unified Parkinson’s Disease Rating Scale; MoCA, Montreal Cognitive Assessment; PD-Left, Parkinson’s disease with left dominant putamen asymmetry; PD-Right, Parkinson’s disease with right dominant putamen asymmetry; SCOPA-AUT, Scales for Outcomes in Parkinson’s Disease-AutonomicDysfunction.

### Speech differences

**Figure 1:**
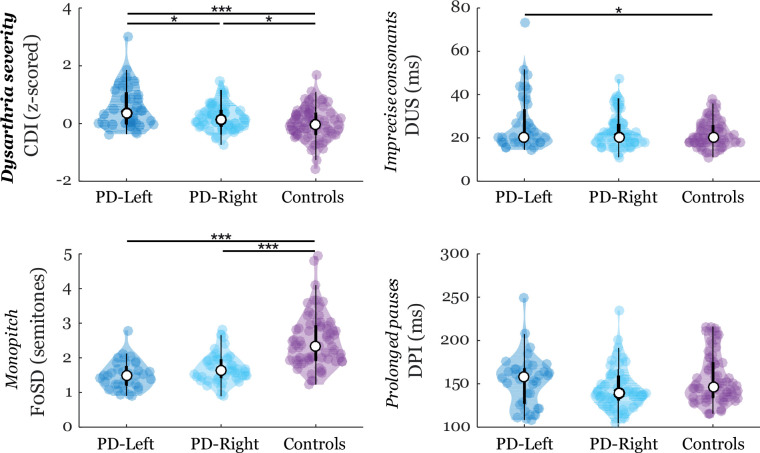
Violin plots of speech characteristics. The plot shows the median (indicated by the black open circle), the first through the third interquartile range (the thick, solid vertical band), an estimator of the density (colour vertical curves) of the individual scores in each group (comparable to a box plot, except that the distribution of the variable is illustrated as density curves) and individual scores (colour filled circles). Statistically significant differences between groups: *p<0.05 and ***p<0.001. CDI, composite dysarthria index; DPI, duration of pause intervals; DUS, duration of unvoiced stops; F0SD, fundamental frequency variability; PD-Left, Parkinson’s disease with left dominant putamen asymmetry; PD-Right, Parkinson’s disease with left dominant putamen asymmetry.

Worse performance in PD-Left compared with the PD-Right group was found for dysarthria severity (CDI: p=0.01) (figure 1); a trend towards significance with worsened performance in the PD-Left group for imprecise consonants (DUS: p=0.08, unadjusted p<0.05) and monopitch (F0SD: p=0.06, unadjusted p<0.05) was also seen. For the entire PD cohort (n=135), a linear negative association between the dysarthria severity and degree of lateralisation in DAT availability towards the left hemisphere was found in putamen (r=−0.22, p=0.01) but not in caudate (online supplemental figure S1).

## Discussion

Based on a large sample of patients with de novo PD and objective acoustic analysis, this study is the first to show that speech is more affected in predominantly right-handed patients with lower left DAT availability in putamen. In contrast, both patient groups showed similar motor and cognitive performance. Previous functional MRI research in healthy speakers revealed activation of different right-sided cortical areas and contralateral subcortical structures, corroborating the assumptions that frontostriatal circuitries play a crucial role in speech production.^12^ In PD, cortical lateralisation effects were also noted, with distinct functional abnormalities observed in the left and right sensorimotor cortex during speech production.^13^

Although language is strongly lateralised to the left hemisphere,^14^ both brain hemispheres are involved in speech production.^15^ This may explain why lateralisation of PD has not been recognised by clinicians in the past. Our understanding of the cortical-subcortical circuits underlying speech control remains limited. Lower DAT availability in the putamen disrupts dopaminergic signalling essential for motor execution, leading to dysarthria via dysfunction in thalamocortical projection. The putamen allows automatic execution of speech as a learnt motor programme, while the thalamus is essential for the coordinated timing of agonist and antagonist muscles participating in speech. Both basal ganglia-thalamic and cerebellothalamic pathways contribute to thalamocortical activation of the motor outflow areas, contributing to fluent speech production. Overall, basal ganglia play a key role in integrating cortical functions within cortico-basal ganglia-thalamo-cortical loops, including cortico-striatal projections from frontal and temporal language areas. Our findings are in line with recent research revealing that dopamine modulates functional connectivity between specific subdivisions of the subthalamic nucleus and language networks.^16^ Surgically targeting subcortical circuits is another promising source of knowledge. Indeed, thalamotomy has been shown to induce dysarthria bilaterally but more frequently in the dominant hemisphere, while dysphasia has been reported after thalamotomy in the dominant hemisphere only.^17^

There was also a trend towards altered consonants and monopitch in patients with lower DAT availability in the left putamen, and altered monopitch appears to be a very early sign of synucleinopathy.^2^ The majority of patients already manifested clinical symptoms and DAT-SPECT abnormalities on both sides. Therefore, we cannot attribute speech impairment solely to the damage of the lower left DAT availability. Indeed, lesions to the right basal ganglia have been documented to cause disturbances in automatic speech production.^18^

In conclusion, our findings raise awareness about hemisphere-specific changes in the speech of patients with PD. Considering the importance of the management of speech following subthalamic deep brain stimulation, the present findings related to asymmetric lateralisation might provide a starting impulse to rethink the way how to programme stimulators to avoid possible side effects of stimulation-induced dysarthria. Future research should also examine the potential laterality of speech disorder earlier in prodromal PD with unilateral DAT-SPECT abnormality presented.

## Supplementary material

10.1136/jnnp-2024-334297 online supplemental file 1
