# Supplementary material for: Is speech function lateralised in the basal ganglia? Evidence from de novo Parkinson’s disease
Source: J Neurol Neurosurg Psychiatry. 2024 Sep 17;96(5):e334297. doi: 10.1136/jnnp-2024-334297 (PMC12015078; doi:10.1136/jnnp-2024-334297)
Supplement: online supplemental file 1 [file jnnp-96-5-s001.pdf]

Figure S1. Correlation between dysarthria severity and (a) asymmetry index of putamen and (b) asymmetry index of caudate.

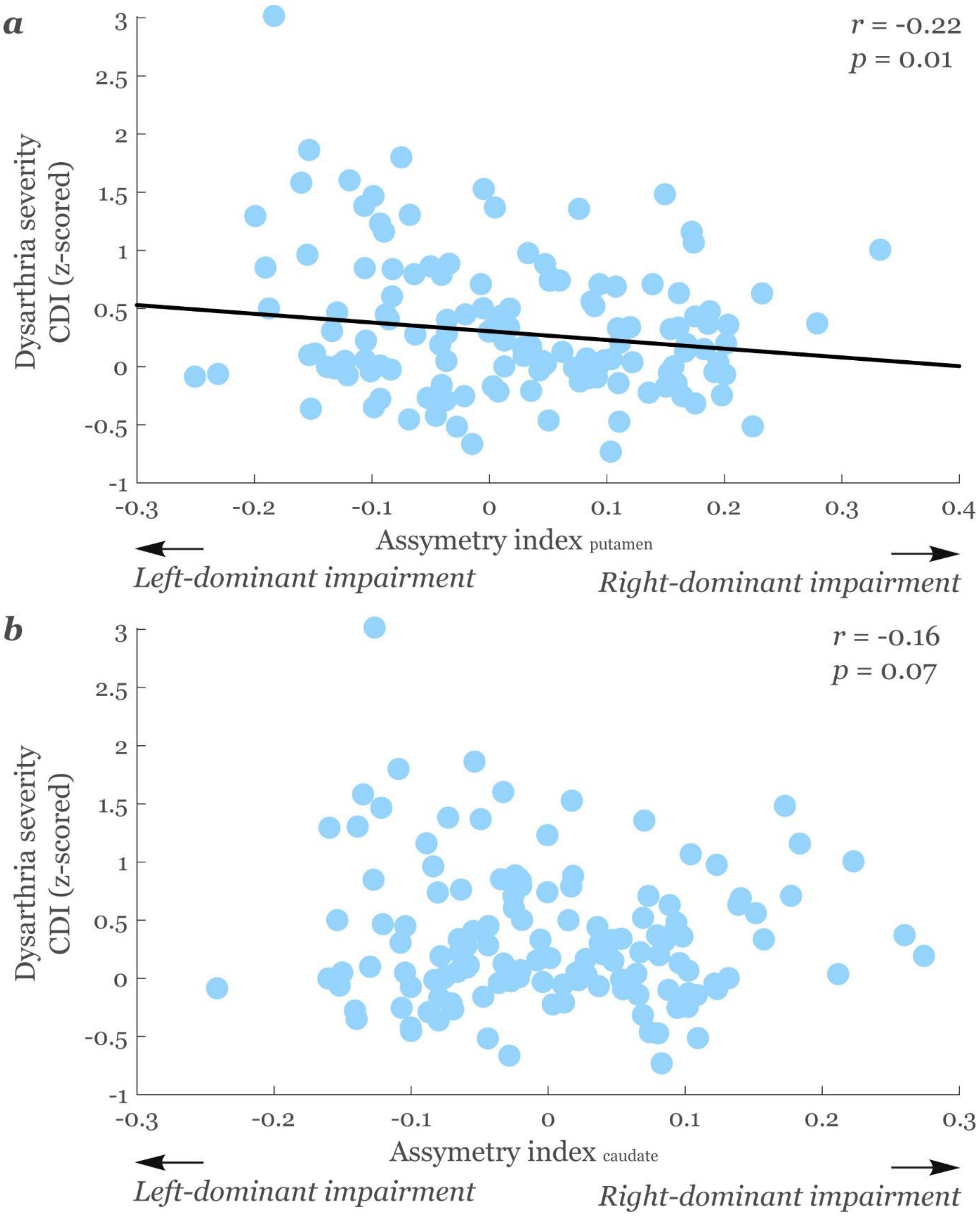

Captions: The blue circles demonstrate the real, uncorrected speech and asymmetry index values, while the correlation coefficient *r* and its corresponding *p*-value are calculated using Pearson's partial correlation analysis controlled for age, sex, and MDS-UPDRS III.
